# Supplementary material for: Hepatic thyroid hormone signalling modulates glucose homeostasis through the regulation of GLP-1 production via bile acid-mediated FXR antagonism
Source: Nat Commun. 2022 Oct 27;13:6408. doi: 10.1038/s41467-022-34258-w (PMC9613917; doi:10.1038/s41467-022-34258-w)
Supplement: Supplementary file 1 — Supplementary information [file 41467_2022_34258_MOESM1_ESM.pdf]

Supplementary information

Hepatic thyroid hormone signalling modulates glucose homeostasis through the regulation of GLP-1 production via bile acid-mediated FXR antagonism

Yan *et al*

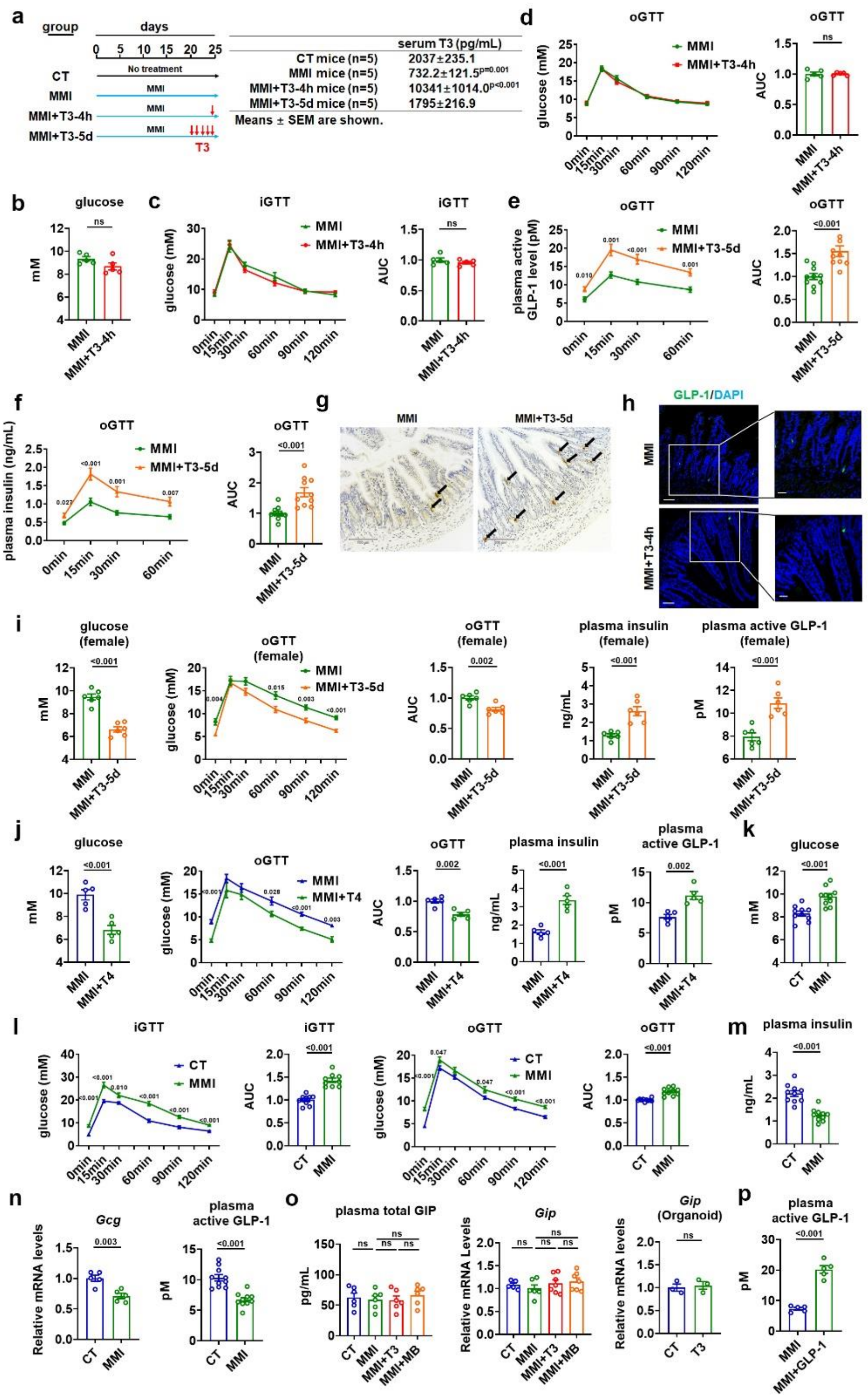
a
group
days
0 5 10 15 20 25
CT
No treatment
MMI
MMI
MMI+T3-4h
MMI
MMI+T3-5d
MMI
T3
serum T3 (pg/mL)
CT mice (n=5) 2037±235.1
MMI mice (n=5) 732.2±121.5 p=0.001
MMI+T3-4h mice (n=5) 10341±1014.0 p<0.001
MMI+T3-5d mice (n=5) 1795±216.9
Means ± SEM are shown.
b glucose
c iGTT
d oGTT
e oGTT
f oGTT
g MMI MMI+T3-5d
h GLP-1/DAPI
MMI
MMI+T3-4h
i glucose (female)
oGTT (female)
plasma insulin (female)
plasma active GLP-1 (female)
j glucose
oGTT
plasma insulin
plasma active GLP-1
k glucose
l iGTT
oGTT
m plasma insulin
n Gcg
plasma active GLP-1
o plasma total GIP
Gip
Gip (Organoid)
p plasma active GLP-1
MMI
MMI+T3-4h
MMI+T3-5d
MMI+T4
CT
MMI+T3
MMI+MB
T3
MMI+GLP-1
Relative mRNA levels
Relative mRNA Levels
glucose (mM)
plasma active GLP-1 level (pM)
plasma insulin (ng/mL)
AUC
mM
ng/mL
pM
pg/mL
ns

Figure S1. Improved metabolism after T3 treatment is attributed to the chronic rather than acute effect of T3.

(a) Schematic diagram of T3 treatment in mice (left). Untreated mice (CT), mice were treated with methimazole (MMI), and then received a single dose of T3 injection (MMI+T3-4h) or daily injection of T3 for 5 days (MMI+T3-5d). The serum T3 levels in CT, MMI, MMI+T3-4h and MMI+T3-5d mice (n=5) (right). (b-d) Blood glucose levels (b), iGTT (c) and oGTT (d) in MMI and MMI+T3-4h mice (n=5). (e and f) Plasma active GLP-1 (e) and insulin (f) levels in MMI and MMI+T3-5d mice during oGTT (left) and the corresponding AUC (right) (n=10). (g) Representative IHC staining of ileal GLP-1 in MMI and MMI+T3-5d mice. Black arrows point to GLP-1 staining. Scale bar: 100 μm. (h) Representative IF staining of ileal GLP-1 (green) and DAPI staining (blue) in MMI and MMI+T3-4h mice. Scale bar (original: 50 μm; enlarged: 20 μm). (i) Blood glucose levels, oGTT and the AUC for oGTT, plasma insulin levels and plasma active GLP-1 levels in female MMI and MMI+T3-5d mice (n=6). (j) Blood glucose levels, oGTT and the AUC for oGTT, plasma insulin levels and plasma active GLP-1 levels in MMI mice and MMI mice treated with T4 for 5 days (n=5). (k-n) Blood glucose levels (n=10) (k), iGTT and the AUC for iGTT (n=9), oGTT and the AUC for oGTT (n=10) (l), plasma insulin levels (n=10) (m), relative mRNA levels of *Gcg* (n=5), plasma active GLP-1 levels (n=10) (n) in CT and MMI mice. (o) Plasma total GIP levels and relative *Gip* mRNA levels in the ileum of CT, MMI, MMI+T3 and MMI+MB mice (n=5-7) and relative *Gip* mRNA levels of mouse intestinal organoids after T3 treatment (n=3 biologically independent experiments). (p) Plasma active GLP-1 levels in MMI mice and MMI mice treated with GLP-1 for 5 days (n=5). iGTT, intraperitoneal GTT; oGTT, oral GTT. Means ± SEM are shown. P values were calculated by two-tailed unpaired Student's t test. ns, not significant. Source data are provided as a Source Data file.

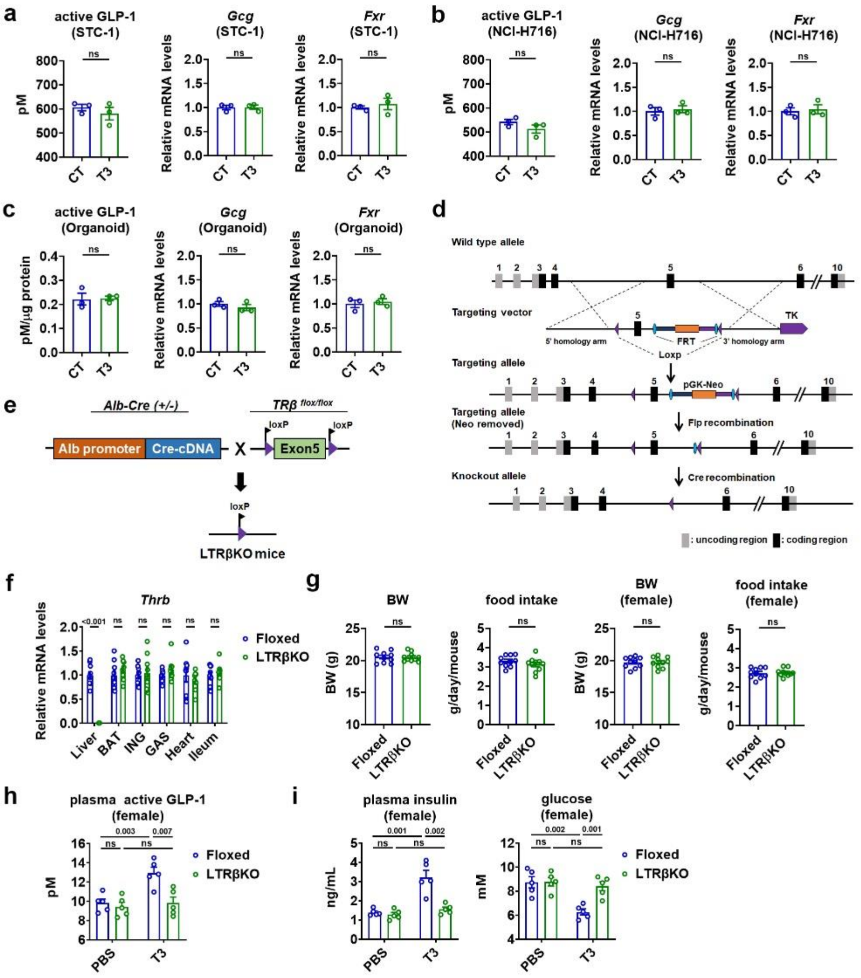

Figure S2. Liver-specific deletion of TRβ is achieved in LTRβKO mice.

(a-c) GLP-1 concentration in the supernatants and relative mRNA levels of *Gcg* and *Fxr* in STC-1 cells (a), NCI-H716 cells (b) and mouse intestinal organoids (c) after T3 treatment (n=3). (d) Schematic diagram of generating a conditional knockout TRβ mouse line that permits the deletion of TRβ in a Cre-recombinase-dependent manner. (e) Breeding scheme for generating LTRβKO mice by crossing TRβ flox/flox mice with transgenic mice expressing Cre-recombinase under the control of albumin promoter (Alb-Cre). (f) Relative mRNA levels of *Thrb* in various tissues (Liver, BAT: brown adipose tissue, ING: inguinal fat, GAS: gastrocnemius muscle, Heart, and Ileum) of Floxed and LTRβKO mice (n=9). (g) The BW and food intake in male and female Floxed and LTRβKO mice as indicated (n=10). (h and i) Plasma active GLP-1 (h), plasma insulin and blood glucose (i) levels in female Floxed and LTRβKO mice treated with PBS or T3 for 5 days as indicated (n=5). Means ± SEM are shown. P values were calculated by two-tailed unpaired Student's t test. ns, not significant. Source data are provided as a Source Data file.

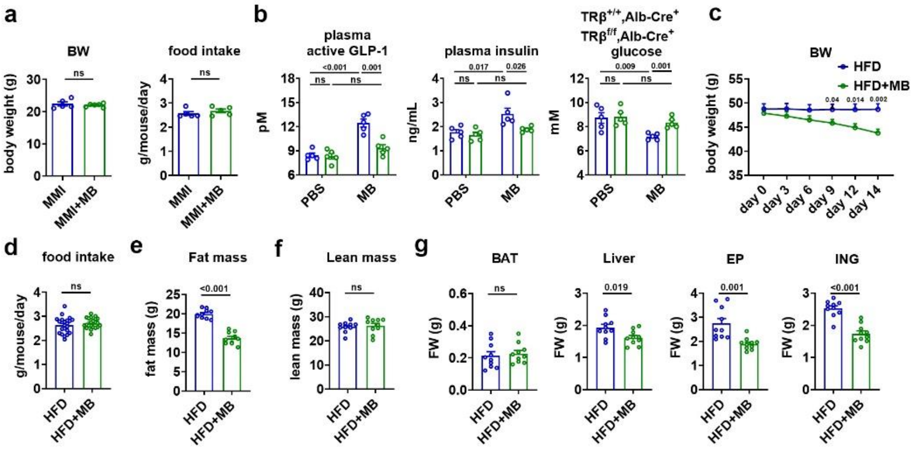

Figure S3. Treatment of MB07811 for 5 days does not affect body weight and food intake of MMI mice.

(a) The body weight (BW) (left) and food intake (right) of MMI mice and MMI mice after 5 days of MB treatment (n=5-6). (b) Plasma active GLP-1, plasma insulin and blood glucose levels in Alb-cre+ control mice (TRβ$^{+/+}$, Alb-cre^{+}) and LTRβKO (TRβ$^{f/f}$, Alb-cre^{+}) mice treated with PBS or MB for 5 days as indicated (n=5). (c and d) The BW curve (c) and food intake (d) of HFD mice treated with or without MB for 14 days (n=10). (e and f) Fat mass (e) and lean mass (f), tissue wight (BAT, Liver, epididymal fat (EP) and inguinal fat (ING)) (g) of HFD mice treated with or without MB for 14 days (n=10). Means ± SEM are shown. P values were calculated by two-tailed unpaired Student's t test. ns, not significant. Source data are provided as a Source Data file.

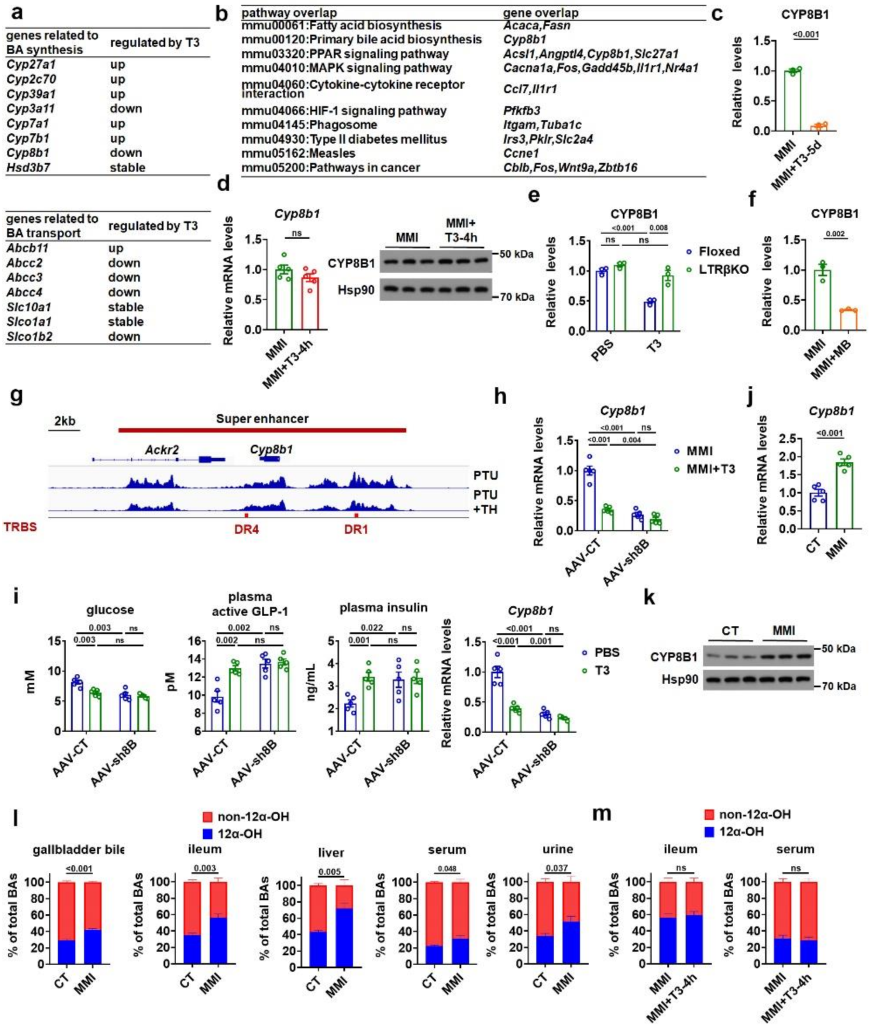

Figure S4. TH shapes the BA composition by targeting CYP8B1 in the liver.

(a) Genes related to BA synthesis and transport regulated by T3 detected by RNA-seq. (b) All DEGs in response to both T3 treatment and food ingestion identified by KEGG and Venn diagram analysis. (c) Densitometry analysis of CYP8B1 protein amounts for the panel e in Figure 4 (n=3). (d) Relative mRNA (left, n=5) and protein (right, n=3) levels of hepatic CYP8B1 in MMI and MMI+T3-4h mice. (e and f) Densitometry analysis of CYP8B1 protein amounts for the panel f (n=3) and g (n=3) in Figure 4. (g) Genome browser snapshot of the mouse *Cyp8b1* locus depicting H3K27ac ChIP-seq coverage in the liver of propylthiouracil (PTU)-treated mice with or without TH treatment and red squares highlight two putative TRβ binding sites (TRBS) (DR1 and DR4) identified by TRβ ChIP-seq (GSE159648, reported by Lazar's lab). The red bar highlights the super-enhancer region predicted in this study. (h) Relative mRNA levels of hepatic *Cyp8b1* in MMI and MMI+T3-5d mice infected with AAV-CT or AAV-sh8B (n=5). (i) Blood glucose, plasma active GLP-1, plasma insulin levels and relative mRNA levels of hepatic *Cyp8b1* in CT and CT+T3-5d mice infected with AAV-CT or AAV-sh8B (n=5). (j and k) Relative mRNA (j, n=5) and protein (k, n=3) levels of hepatic CYP8B1 in CT and MMI mice. (l and m) Relative levels of 12α-OH (blue) and non-12α-OH (red) BAs in the gallbladder bile, ileum, liver, serum, and urine of CT and MMI mice (l, n=5) and in the ileum and serum of MMI and MMI+T3-4h mice (m, n=5). Means ± SEM are shown. P values were calculated by two-tailed unpaired Student's t test. ns, not significant. Source data are provided as a Source Data file.

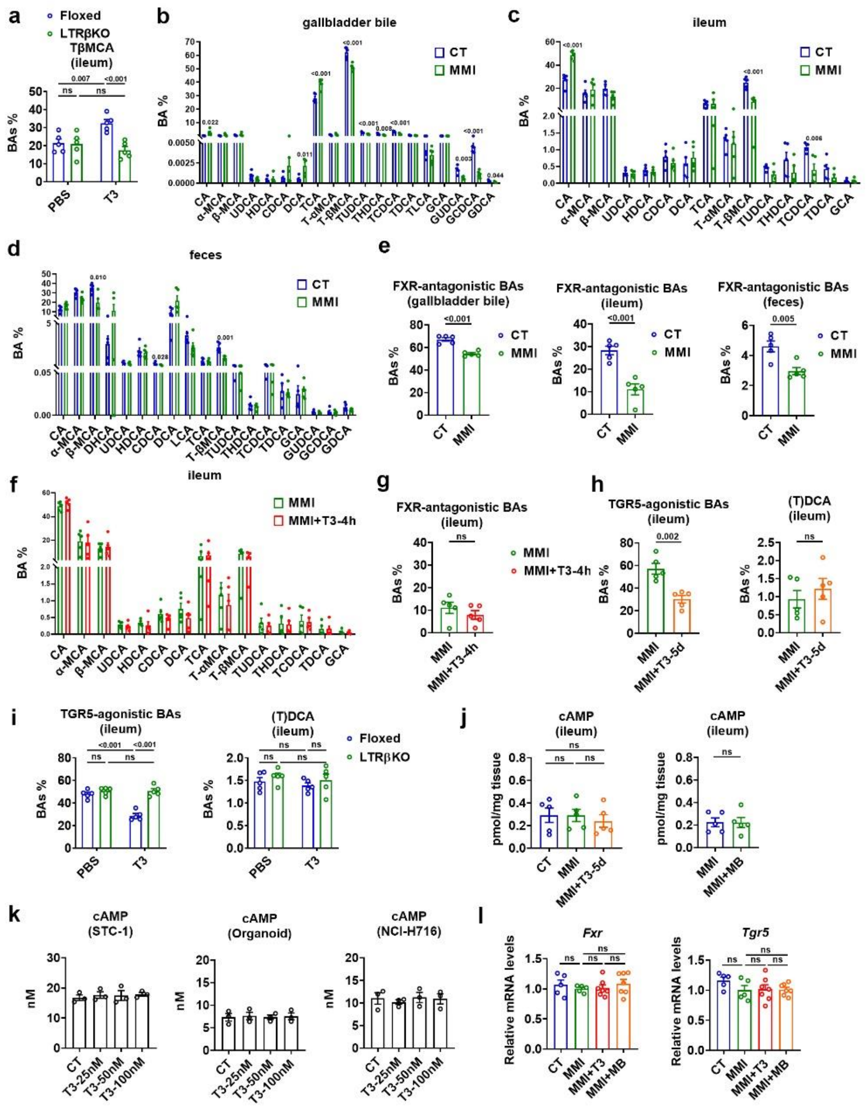

Figure S5. TH potentiates the production of non-12α-OH FXR-antagonistic BAs.

(a) The percentage of TβMCA in the ileum of Floxed and LTRβKO mice treated with PBS or T3 for 5 days (n=5). (b-e) The percentage of individual BA in the gallbladder bile (b), ileum (c) and feces (d) and the percentage of FXR-antagonistic BAs (T(α/β)MCA, (T/G)UDCA and (T)HDCA) in these specimens (e) of CT and MMI mice (n=5). (f and g) The percentage of individual BA (f) and FXR-antagonistic BAs (g) in the ileum of MMI and MMI+T3-4h mice (n=5). (h) The percentage of TGR5-agonistic BAs ((T/G)CA, (T)CDCA, and (T)DCA) in the ileum of MMI and MMI+T3-5d mice (left, n=5). The percentage of (T)DCA in the ileum of MMI and MMI+T3-5d mice (right, n=5). (i) The percentage of TGR5-agonistic BAs (left) and (T)DCA (right) in the ileum of Floxed and LTRβKO mice treated with PBS or T3 for 5 days (n=5). (j) Ileum cAMP levels in CT mice and MMI mice with or without 5 days of T3 (left) or MB (right) treatment as indicated (n=5). (k) cAMP levels in STC-1 cells, mouse intestinal organoids, and NCI-H716 cells treated with an increasing dose of T3 as indicated for 2 h (n=3). (l) Relative mRNA levels of *Fxr* (left) and *Tgr5* (right) in the ileum of CT mice (n=5), MMI mice (n=5), and MMI mice after 5 days of T3 (n=7) or MB (n=7) treatment as indicated. Means ± SEM are shown. P values were calculated by two-tailed unpaired Student's t test. ns, not significant. Source data are provided as a Source Data file.

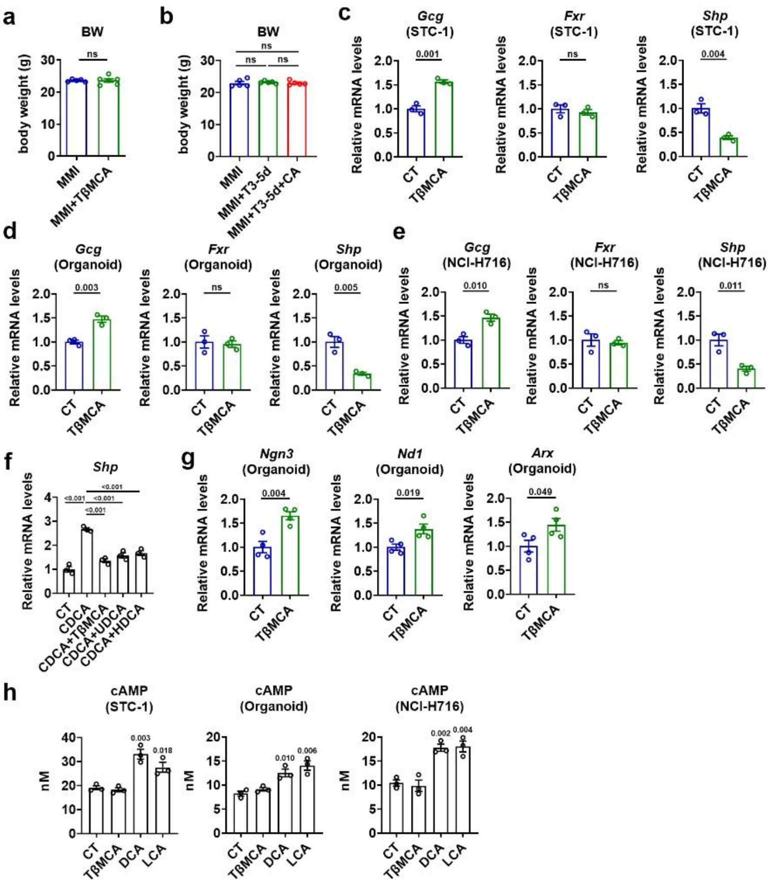

Figure S6. Non-12α-OH FXR-antagonistic BAs-mediated the glucose-lowering effect of T3 is independent of weight loss.

(a and b) The BW of MMI mice with or without 5 days of TβMCA treatment (a, n=5-6) and the BW of MMI mice treated with T3 and CA for 5 days as indicated (b, n=5). (c-e) Relative mRNA levels of *Gcg*, *Fxr* and *Shp* in STC-1 cells, mouse intestinal organoids and NCI-H716 cells after TβMCA treatment (n=3). (f) Relative mRNA levels of *Shp* in NCI-H716 cells treated with CT, or CDCA alone or together with other FXR-antagonistic BAs as indicated (n=3). (g) Relative mRNA levels of *Ngn3*, *Nd1*, and *Arx* in mouse intestinal organoids treated with TβMCA (n=4). (h) cAMP levels in STC-1 cells, mouse intestinal organoids and NCI-H716 cells treated with TβMCA, DCA, and LCA (n=3). Means ± SEM are shown. P values were calculated by two-tailed unpaired Student's t test. ns, not significant. Source data are provided as a Source Data file.

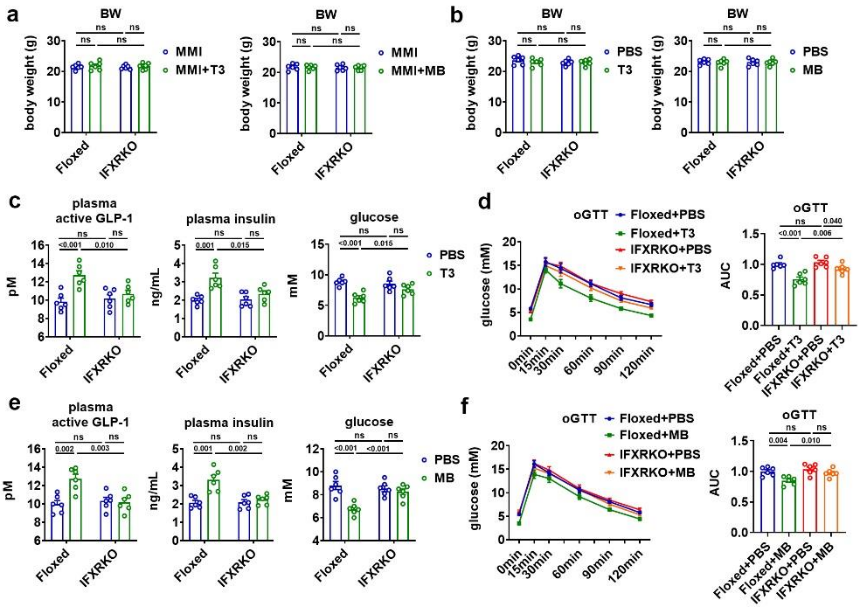

Figure S7. Intestinal FXR mediates the hepatic T3 effect on the GLP-1 production and glucose homeostasis.

(a) The BW of Floxed and IFXRKO mice treated with MMI or MMI and 5 days of T3 or MB as indicated (n=6). (b) The BW of Floxed and IFXRKO mice treated with 5 days of T3 or MB as indicated (n=6). (c) Plasma active GLP-1, plasma insulin, and blood glucose levels in Floxed and IFXRKO mice treated with PBS or T3 for 5 days (n=6). (d) oGTT for Floxed and IFXRKO mice treated with treated with PBS or T3 for 5 days (left) and the AUC for oGTT (right) (n=6). (e) Plasma active GLP-1, plasma insulin, and blood glucose levels in Floxed and IFXRKO mice treated with PBS or MB for 5 days (n=6). (f) oGTT for Floxed and IFXRKO mice treated with treated with PBS or MB for 5 days (left) and the AUC for oGTT (right) (n=6). Means ± SEM are shown. P values were calculated by two-tailed unpaired Student’s t test. ns, not significant. Source data are provided as a Source Data file.

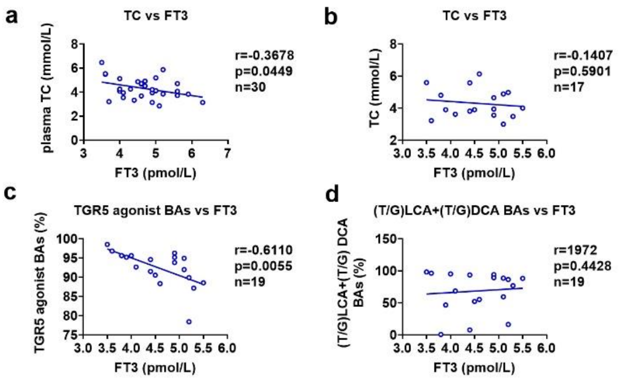

Figure S8. The relationship among TH, TSH, GLP-1, insulin, cholesterol, and BA levels in people with normal thyroid function.

(a) Correlation between free T3 levels and plasma total cholesterol (TC) levels in a cohort of euthyroid human study participants (n=30). (b-d) Correlation between free T3 levels and TC levels (b, n=17), between free T3 levels and the percentage of fecal TGR5-agonistic BAs, including (T/G)CA, (T/G)CDCA, (T/G)DCA and (T/G)LCA (c, n=19), or between free T3 levels and the percentage of fecal (T/G)DCA and (T/G)LCA (d, n=19) in another cohort of euthyroid human study participants. Pearson correlation and two-tailed t-test was performed for correlation analysis. Source data are provided as a Source Data file.

**Number**	**30**
**Gender**	
**Male**	**8/30 (26.7%)**
**Female**	**22/30 (73.3%)**
**Age (year)**	**36.30±2.07**
**FT3 (pmol/L)**	**4.67±0.13**
**FT4 (pmol/L)**	**16.96±0.30**
**TSH (pmol/L)**	**2.40±0.26**

Supplementary Table 1. The information of human study participants for GLP-1 measurement. Means ± SEM are shown.

**Number**	**19**
**Gender**	
**Male**	**5/19 (26.3%)**
**Female**	**14/19 (73.7%)**
**Age (year)**	**44.37±3.64**
**FT3 (pmol/L)**	**4.57±0.14**
**FT4 (pmol/L)**	**16.63±0.47**
**TSH (pmol/L)**	**2.15±0.23**

Supplementary Table 2. The information of human study participants for bile acid measurement. Means ± SEM are shown.

Primer	Sequence	Note
***18s* F**	**ACCGCAGCTAGGAATAATGGA**	**RT-PCR**
***18s* R**	**CAAATGCTTTCGCTCTGGTC**	**RT-PCR**
**m*Arx* F**	**GTTACCAGCTGGAGGAACTG**	**RT-PCR**
**m*Arx* R**	**GGCCTCTGTCAGGTCCAG**	**RT-PCR**
**m*Cyp27a1* F**	**AGGGCCTCACATCAACAGAG**	**RT-PCR**
**m*Cyp27a1* R**	**GCTGACGCTGTAGGACACAT**	**RT-PCR**
**m*Cyp7a1* F**	**GAGAGTGAATCAGGGGACCA**	**RT-PCR**
**m*Cyp7a1* R**	**TCAGGAATGGAGGGTTTCAG**	**RT-PCR**
**m*Cyp7b1* F**	**GGAGCCACGACCCTAGATG**	**RT-PCR**
**m*Cyp7b1* R**	**TGCCAAGATAAGGAAGCCAAC**	**RT-PCR**
**m*Cyp8b1* F**	**GGGAGTGGGTGGAAGTGAG**	**RT-PCR**
**m*Cyp8b1* R**	**GTCCTGCATGGATGAAGCT**	**RT-PCR**
**m*Fxr* F**	**TGGGCTCCGAATCCTCTTAGA**	**RT-PCR**
**m*Fxr* R**	**TGGTCCTCAAATAAGATCCTTGG**	**RT-PCR**
**m*Gip* F**	**GAGTTCCGATCCCATGCTAA**	**RT-PCR**
**m*Gip* R**	**TTGTTGTCGGATCTTGTCCA**	**RT-PCR**
**m*Glut2* F**	**TGGCTGCCTTCAGCAACTG**	**RT-PCR**
**m*Glut2* R**	**CAAGGAAGTCCGCAATGTACTG**	**RT-PCR**
**m*Nd1* F**	**AGGTGGTACCTTGCTACTCC**	**RT-PCR**
**m*Nd1* R**	**TGAAAGAGAAGTTGCCATTG**	**RT-PCR**
**m*Ngn3*F**	**GCATGCACAACCTCAACTC**	**RT-PCR**
**m*Ngn3* R**	**TTTGTAAGTTTGGCGTCATC**	**RT-PCR**
**m*Gcg* F**	**GATCATTCCCAGCTTCCCAG**	**RT-PCR**
**m*Gcg* R**	**CTGGTAAAGGTCCCTTCAGC**	**RT-PCR**
**m*Pyy* F**	**GCAGCGGTATGGAAAAAGAG**	**RT-PCR**
**m*Pyy* R**	**GTCGCTGTCGTCTGTGAAGA**	**RT-PCR**
**m*Sglt1* F**	**TGGTGTACGGATCAGGTCATTG**	**RT-PCR**
**m*Sglt1* R**	**TCCAGATAGCCACACAGGGTACAG**	**RT-PCR**
**m*Shp* F**	**CCGACTATTCTGTATGCACTTC**	**RT-PCR**
**m*Shp* R**	**TCCTGTTGCAGGTGTGCG**	**RT-PCR**
**m*Tgr5* F**	**GCTGCTCACAGGGCT**	**RT-PCR**
**m*Tgr5* R**	**AACACGCGGACAGAGAG**	**RT-PCR**
**m*Thrb* F**	**CAGCCTGGGACAAACAGAAG**	**RT-PCR**
**m*Thrb* R**	**GGTGACTTTGTCTATGATGC**	**RT-PCR**
**h*Fxr* F**	**GGAGACAGAGCCTCTGGA**	**RT-PCR**
**h*Fxr* R**	**TTCTCCCTGCATGACTTTGTT**	**RT-PCR**
**h*Gip* F**	**CCTTTGCTCTGCTGCTG**	**RT-PCR**
**h*Gip* R**	**CTTCTTCCCCTTTTGGGCC**	**RT-PCR**
**h*Glut2* F**	**ACACACTTGGAAGAATCAAAGC**	**RT-PCR**
**h*Glut2* R**	**GAAAAAGAGTAGCAGAGACTGAAG**	**RT-PCR**
**h*Gcg* F**	**TGGCTGGATTATTTGTAATGCTGGTA**	**RT-PCR**
**h*Gcg* R**	**ACCTCTTCTGGGAAATCTCG**	**RT-PCR**
**h*Pyy* F**	**TTCTCCCTGCATGACTTTGTT**	**RT-PCR**
**h*Pyy* R**	**CGCCGTCGGGGAAGAA**	**RT-PCR**
**h*Sglt1* F**	**CCCCATCTATATTAAGGCTGGG**	**RT-PCR**
**h*Sglt1* R**	**GAATGGCTTTCATGTACTTTTCCA**	**RT-PCR**
**h*Shp* F**	**AAGACAGTGGCCTTCCTC**	**RT-PCR**
**h*Shp* R**	**GGGTTGAAGAGGATGGTCCC**	**RT-PCR**

**h*Tgr5* F**	**CAGGGCTGTGGAACCAG**	**RT-PCR**
**h*Tgr5* R**	**CCATAGACTTCGAGGTACAGG**	**RT-PCR**
**h*Actb* F**	**GATCATTGCTCCTCCTGAGC**	**RT-PCR**
**h*Actb* R**	**ACTCCTGCTTGCTGATCCAC**	**RT-PCR**
**DR1 1-F**	**AGACCYACAAAGGCCAGT**	**CHIP**
**DR1 1-R**	**AGCAACATATATGCTATGTGTACTT**	**CHIP**
**DR4 1-F**	**CTCAGCACACTTGTCCG**	**CHIP**
**DR4 1-R**	**CCTGCCCACCAATCCT**	**CHIP**

Supplementary Table 3. Primer information.
