# Supplementary material for: Hepatic thyroid hormone signalling modulates glucose homeostasis through the regulation of GLP-1 production via bile acid-mediated FXR antagonism
Source: Nat Commun. 2022 Oct 27;13:6408. doi: 10.1038/s41467-022-34258-w (PMC9613917; doi:10.1038/s41467-022-34258-w)
Supplement: Supplementary file 3 — Description of Additional Supplementary Files [file 41467_2022_34258_MOESM3_ESM.docx]

Description of Additional Supplementary Files

File Name: Supplementary Data 1
Description: The concentration of individual BA in the gallbladder bile, liver, ileum, serum, feces and urine of CT, MMI and MMI+T3-5d mice.

File Name: Supplementary Data 2.
Description: The concentration of individual BA in the ileum of Floxed and LTRβKO mice treated with PBS or T3 for 5 days.

File Name: Supplementary Data 3
Description: The concentration of individual BA in the feces of a cohort of euthyroid human study participants.
